# Potent Neutralization Ability of a Human Monoclonal Antibody Against Serotype 1 Dengue Virus

**DOI:** 10.3389/fmicb.2018.01214

**Published:** 2018-06-06

**Authors:** Jiansheng Lu, Rong Wang, Binghui Xia, Yunzhou Yu, Xiaowei Zhou, Zhixin Yang, Peitang Huang

**Affiliations:** Laboratory of Protein Engineering, Beijing Institute of Biotechnology, Beijing, China

**Keywords:** human monoclonal antibody, 1G5, dengue virus serotype 1, envelope protein, antibody-dependent enhancement, antibody neutralization, single plasma cells

## Abstract

The incidence of dengue virus (DENV) infections has been escalating in tropical and subtropical countries, but there are still no effective therapeutic options. In the present study, a DENV-1-specific human monoclonal antibody (HMAb), 1G5, isolated from single plasma cells obtained from the peripheral blood mononuclear cells of dengue patients was found to have potent neutralization activity against serotype 1 DENV (DENV-1). Its neutralization activity against DENV-2 was not as strong, and it was almost absent for DENV-3 and DENV-4. The results showed that HMAb 1G5 only binds to the envelop protein of intact DENV-1 or the envelop protein under unheated and non-reducing conditions, and that it does not bind to recombinant envelope protein. This could mean that the antibody recognizes a conformational epitope of the envelope protein. Further, the findings showed that HMAb 1G5 potently neutralizes DENV-1 in both the pre- and post-attachment phases of the virus at low concentrations. *In vivo* studies showed that HMAb 1G5 provides protection from DENV-1 infection in a murine model. In addition, antibody-dependent enhancement that occurs at lower doses of the antibody was completely abrogated by the introduction of Leu-to-Ala mutations (1G5-LALA) or deletion of nine amino acids (1G5-9del) in the Fc region. Therefore, HMAb 1G5 shows promise as a safe and effective agent for prophylactic and therapeutic treatment of DENV-1 infection.

## Introduction

Dengue virus (DENV), which belongs to the Flaviviridae family, is an important arthropod-borne virus that targets humans. The DENV particle contains an RNA genome that is a single sense strand (11 kb) and forms a complex with a capsid protein. A bilayer lipid membrane envelopes the nucleocapsid; the membrane is composed of 180 copies each of the envelope (E) and membrane (M) proteins (Kuhn et al., 2002; Zhang et al., 2013). Crystal structure analysis revealed that the E protein contains three domains, namely, DI, DII, and DIII, and that it has an essential function in the attachment and fusion of DENV at the time of its entry into the host (Modis et al., 2003, 2004, 2005; Zhang et al., 2004; Chin et al., 2007; Klein et al., 2013). Infection caused by one of the four DENV serotypes (DENV-1–4) results in the manifestation of symptoms such as mild dengue fever, serious dengue hemorrhagic fever, and dengue shock syndrome; in addition, some of the affected patients are also asymptomatic (Halstead, 2007; Simmons et al., 2012). DENV infection is mainly prevalent in tropical and sub-tropical regions, with the highest incidence rates reported in the Americas, Eastern Mediterranean, Southeast Asia, and the Western Pacific regions. Sometimes, different serotypes of DENV can cause epidemics in different regions of the world at the same time. The circulation of DENV-1 and DENV-2 serotypes in the Western Pacific region was reported in 2017^1^. Most epidemics are caused by a single serotype, but others are caused by two or more serotypes (Morens et al., 1986). All four serotypes of DENV can cause epidemics in the same region; for example, in Guangdong Province, China, there has been outbreak of all four serotypes of DENV, although DENV-1 was the predominant strain (Wu et al., 2011; Jiang et al., 2012; Zhao et al., 2012a,b). The worldwide incidence of DENV infection has seen a considerable increase in the last few decades. Globally, about 400 million DENV cases are detected every year: the number of reported cases is 100 million and the number of associated deaths is about 20,000 (Thomas and Endy, 2011; Bhatt et al., 2013). Dengue is also a threat in southern China, where 46864 cases and six associated deaths were reported in 2014^2^.

Sanofi-Pasteur released the live-attenuated tetravalent vaccine Dengvaxia based on the chimeric yellow fever-DENV in 2015 (Hadinegoro et al., 2015). Dengvaxia is now available across a few countries. Unfortunately, when Dengvaxia is used in DENV-negative individuals, disease severity increases when they acquire the infection later. Therefore, its use is mostly limited to adults from endemic countries. In fact, this vaccine is now out of use in the Philippines. Therefore, there is a need for effective therapeutic treatments against this virus. Therapeutic treatment with antibodies is now emerging as a potential treatment method against viral infections. Among the neutralizing antibodies that are currently under research, the most successful monoclonal antibody identified is palivizumab, which is a humanized mouse monoclonal antibody that was found to significantly reduce the risk of hospitalization due to RSV infection based on clinical trials in children (Kliks et al., 1988; Chau et al., 2008). For the treatment of dengue, it has been reported that serotype-specific antibodies have the strongest neutralization capacity *in vitro* settings (Sukupolvi-Petty et al., 2007; Gromowski et al., 2008; de Alwis et al., 2012; Teoh et al., 2012). Moreover, passive transfer of monoclonal or polyclonal antibodies has been shown to have a protective effect against homologous or heterologous DENV challenge in mice (Goncalvez et al., 2004; Kyle et al., 2008). Several panels of DENV-specific human monoclonal antibodies (HMAbs) have recently been generated with memory B cells or plasma cells obtained from patients who were vaccinated or naturally acquired DENV infection; these panels have been used to study the humoral immune response to dengue (Beltramello et al., 2010; de Alwis et al., 2012; Smith et al., 2012, 2013a,b, 2014; Teoh et al., 2012; Costin et al., 2013; Tsai et al., 2013; Dejnirattisai et al., 2015). The main cross-reactive HMAbs were obtained from memory B cells or plasma cells of patients after they had primary or secondary infection, but type-specific HMAbs were mainly obtained from patients after primary infection.

In the present study, we used fluorescence-activated cell sorting (FACS) to isolate single plasma cells from peripheral blood mononuclear cells (PBMCs) sampled from dengue patients from Guangdong Province who had naturally acquired dengue infection in 2014. Gene amplification was performed using single-cell PCR to generate a panel of HMAbs against DENV. From this panel, we identified HMAb 1G5 as a potent antibody that functions effectively against DENV-1 under *in vitro* and *in vivo* settings at low concentrations. Further, antibody-dependent enhancement (ADE) was abrogated without any compromise on the efficacy of the antibody, which eliminates concerns about the safety of this dengue therapeutic antibody.

## Materials and Methods

### Cell Lines and Viruses

C6/36 cells and K562 cells (ATCC) were cultured in RPMI-1640 medium containing 10% fetal bovine serum (FBS, Excell). BHK-21 (ATCC) cells were cultured in Dulbecco modified Eagle medium (DMEM) containing 10% FBS. FreeStyle^TM^ 293-F cells (Invitrogen) were cultured in FreeStyle 293 Expression Medium (12338; Gibco). C6/36 cells were incubated at 28°C in the absence of CO_2_, and the other cells were incubated at 37°C in a 5% CO_2_ atmosphere. The GE27 strain of DENV-1, the New Guinea C (NGC) strains of DENV-2, the YN01 strain of DENV-3, and the 30 strain of DENV-4 used in the experiments were sourced from our laboratory. Culture supernatant of the infected cells was centrifuged at 2000 *g* to get rid of cell debris, and the virus fraction obtained was aliquoted and stored at -80°C.

### Isolation of Single Human Plasma Cells and HMAb Generation

As shown in **Table 1**, three patients from Guang Dong province of China who had naturally acquired DENV infection in 2014 was sampled. DENV-1 infection was confirmed by testing for the presence of neutralizing antibodies against DENV-1. PBMCs were obtained by Ficoll density gradient separation. DENV-specific HMAbs were generated from plasma cells (Wrammert et al., 2008, 2012; Smith et al., 2009). The PBMCs obtained were stained with FITC mouse anti-human CD3 (555332, BD Pharmingen), APC Mouse Anti-Human CD19 (555415, BD Pharmingen), FITC mouse anti-human CD20 (555622, BD Pharmingen), PE mouse anti-human CD27 (555441, BD Pharmingen), and PE-Cy^TM^7 mouse anti-human CD38 (560677, BD Pharmingen) antibodies. Following this, the activated antibody-secreting cells were classified as CD19^high^ CD3^negative^ CD20^low to negative^ CD27^high^ CD38^high^. The cells were sorted into 96-well PCR plates containing RNase inhibitor (N2611, Promega), with each well containing a single antibody-secreting cell. The PCR plates were centrifuged at 100 *g* for 5 min before they were frozen on dry ice and stored at -80°C. RT-PCR (210212, Qiagen) and nested PCR (AP141, Transgen) were performed on the H-chain, λ-chain, and κ-chain genes. Primer cocktails that were specific to IgG were used [**Table 2** (Smith et al., 2009) and **Table 3**]. The amplicon size was 350–400 bp for all the antibody chains. The PCR products obtained were digested with the restriction endonucleases Sal-I and Pml-I, and cloned into separate plasmid vectors pTSEG1n, pTSEK, or pTSEL (all of which were constructed by our lab and the constructs were shown in **Supplementary Figure S1**) containing a human immunoglobulin constant gene. The recombinant antibodies were generated as IgG1 molecules, irrespective of their original isotype. For the expression of antibodies, FreeStyle^TM^293-F cells were transfected with equal parts of plasmids encoding heavy and light chains using the FectoPRO^®^ transfection kit (116-001, Polyplus-transfection). Four days later, antibody-containing supernatants were harvested and purified using HiTrap MabSelect Xtra (28-4082-60, GE Healthcare).

**Table 1:** Information on dengue patients enrolled in the study.

Patient No.	Gender	Age	Days after illness onset
1	Female	34	15
2	Female	28	16
3	Male	19	16

**Table 2:** Primer sequences for RT-PCR (Smith et al., 2009).

Primer	Sequence
5′ L-VH 1	ACAGGTGCCCACTCCCAGGTGCAG
5′ L-VH 3	AAGGTGTCCAGTGTGARGTGCAG
5′ L-VH 4/6	CCCAGATGGGTCCTGTCCCAGGTGCAG
5′ L-VH 5	CAAGGAGTCTGTTCCGAGGTGCAG
HuIgG-const-anti	TCTTGTCCACCTTGGTGTTGCT
3′ Cμ CH1	GGGAATTCTCACAGGAGACGA
5′ L Vκ 1/2	ATGAGGSTCCCYGCTCAGCTGCTGG
5′ L Vκ 3	CTCTTCCTCCTGCTACTCTGGCTCCCAG
5′ L Vκ RT-PCR	ATTTCTCTGTTGCTCTGGATCTCTG
3′ Cκ 543–566	GTTTCTCGTAGTCTGCTTTGCTCA
5′ L Vλ 1	GGTCCTGGGCCCAGTCTGTGCTG
5′ L Vλ 2	GGTCCTGGGCCCAGTCTGCCCTG
5′ L Vλ 3	GCTCTGTGACCTCCTATGAGCTG
5′ L Vλ 4/5	GGTCTCTCTCSCAGCYTGTGCTG
5′ L Vλ 6	GTTCTTGGGCCAATTTTATGCTG
5′ L Vλ 7	GGTCCAATTCYCAGGCTGTGGTG
5′ L Vλ 8	GAGTGGATTCTCAGACTGTGGTG
3′ Cλ	CACCAGTGTGGCCTTGTTGGCTTG

**Table 3:** Part of primer sequences for nested PCR.

Primer	Sequence
5′ VH1	CAGRTGCAGCTGGTGCARTCTGG
5′ VH2	SAGGTCCAGCTGGTRCAGTCTGG
5′ VH3	CAGRTCACCTTGAAGGAGTCTGG
5′ VH4	SAGGTGCAGCTGGTGGAGTCTGG
5′ VH5	GAGGTGCAGCTGGTGGAGWCYGG
5′ VH6	CAGGTGCAGCTACAGCAGTGGGG
5′ VH7	CAGSTGCAGCTGCAGGAGTCSGG
5′ VH8	GARGTGCAGCTGGTGCAGTCTGG
5′ VH9	CAGGTACAGCTGCAGCAGTCAGG
3′ VHR1	GGCCCTTGGTGCTAGCTGAGGAGACGGTGACCAGGGTKCC
3′ VHR2	GGCCCTTGGTGCTAGCTGAAGAGACGGTGACCATTGTCCC
3′ VHR3	GGCCCTTGGTGCTAGCTGAGGAGACGGTGACCGTGGTCCC

### Neutralization Potency of all DENV-Specific HMAbs Against DENV-1 and Neutralization Efficiency of HMAb 1G5 Against All Four Serotypes DENV

BHK-21 cells were seeded (5 × 10^5^ cells/ml) in each well (containing 2 ml of the medium) of a 6-well plate, which was incubated overnight at 37°C in a 5% CO_2_ atmosphere. The BHK-21 cell monolayers that had formed in the plates were treated with the antibody-virus suspension, and they were incubated for another 1 h at 37°C. The culture supernatant was discarded, and 2 ml of 1.0% (w/v) LMP agarose (0815, Amresco) in DMEM containing 2% (v/v) FBS was added. This was followed by incubation for 4–5 days at 37°C, after which the cells were fixed with 4% (v/v) formaldehyde by incubation at 4°C for 1 h. Finally, the cells were stained with 0.1% (w/v) crystal violet by incubation at RT for 30 min to observe plaque formation.

To evaluate the neutralization potency of the DENV-specific HMAbs against DENV-1, the concentration of all the antibodies was fixed at 10 μg/ml. Then each antibody was added to approximately 100 PFU of DENV-1 and incubated at 37°C for 1 h before it was added to the BHK-21 cell monolayers. The number of plaques formed by DENV-1 after treatment with each antibody was quantified to determine the neutralization potency of the antibodies.

To evaluate the neutralization efficiency of HMAb 1G5 against all four DENV serotypes, three-fold serial dilutions of HMAb 1G5 were added to all four serotypes of DENV (approximately 100 PFU) and incubated at 37°C for 1 h before they were added to the BHK-21 cell monolayers. PRNT_50_ represents the antibody concentration at which there is a 50% reduction in plaque formation. PRNT_50_ was calculated with the help of a non-linear curve fit in GraphPad Prism

### Immunoblot Analysis

DENV-containing culture supernatants were obtained from C6/36 cells and divided into two groups. The reducing group was added with loading buffer containing 100 mM beta-mercaptoethanol while the unheated and non-reducing group was added with loading buffer without beta-mercaptoethanol. The samples were then separated using 10% sodium dodecyl sulfate-polyacrylamide gel electrophoresis and transferred to polyvinylidene fluoride membranes (10600023; Amersham) by electroblotting. Non-specific binding was blocked using 5% skimmed milk, after which the membranes were treated with DENV-specific HMAb 1G5 (1 μg/ml) and then horseradish peroxidase-conjugated antibody against human IgG (1:5000, v/v). The membranes were then treated with an enhanced chemiluminescence substrate (RPN2106, Amersham). DV69.6 (1 μg/ml) (Beltramello et al., 2010), which is a cross-reactive antibody against DENV, recognizes the prM protein and is an indicator of the molecular weight of prM. 4G2 (1 μg/ml) (Henchal et al., 1985), which is a cross-reactive antibody against flavivirus, recognizes a conserved epitope that is present on the fusion loop of the E protein; it is an indicator of the molecular weight of the E protein.

### ELISA Assay

To examine whether HMAb 1G5 is specific to any of the DENV serotypes, we performed ELISA with the intact virion and the recombinant E (rE) protein. For virion ELISA, assay plates (9018, Costar) were coated with the 4G2 antibody 200 ng per well in order to capture the virion present in the culture supernatant of infected C6/36 cells. In rE ELISA, the assay plates were coated with the rE proteins of the four DENV serotypes 200 ng per well (228-11688, 228-11689, 228-11690, 228-11691; RayBiotech) expressed by *Escherichia coli*.

The ELISA plates were coated overnight at 4°C, and then the plates were blocked by incubation with 200 μl of 5% (w/v) skimmed milk-PBS in each well for 2 h at 37°C. HMAb 1G5 was serially diluted in blocking buffer from an initial concentration of 30 μg/ml, and then 100 μl of each concentration was added to each well and incubated for 2 h at 37°C. HMAb S4B11 (which is an antibody generated by us) recognizes PDL-1 and was used as a non-related control to bind the rE proteins. HMAb 6B1 (which is an antibody also generated by us) recognizes the E protein of all four DENV serotypes and was used as a positive control. The plates were then washed three times with PBS-Tween (0.1% v/v). Next, goat anti-human horseradish peroxidase-conjugated IgG antibody (1:5000, v/v) was added for 1 h at 37°C. This was followed by three rounds of washing with PBS-Tween, and finally, an OPD chromogen substrate was used for detection at OD492 nm.

### Pre- and Post-attachment Neutralization Efficiency of HMAb 1G5

BHK-21 cells in 6-well plates and the reagents used were cooled to 4°C. The cells were incubated with serially diluted HMAb 1G5 1 h before (pre) or after (post) DENV virus samples were added to the cells at 4°C. If the cells were primarily treated with the virus, any unbound virus was washed off by washing twice with cold PBS. If the cells were primarily incubated with the antibody, virus samples were directly added without removal of the antibody. After incubation again for 1 h at 4°C, the cell monolayers were washed three times with cold PBS, and 2 ml of 1.0% (w/v) LMP agarose in DMEM containing 2% FBS was added. The cells were further incubated at 37°C for 4 days, after which they were fixed and stained for observation of plaque formation, as described in the previous subsection.

### *In Vivo* Protective Efficacy of HMAb 1G5

The four DENV serotypes were obtained from brain suspensions of suckling mice with DENV infection. One-day-old Balb/c suckling mice were intracerebrally inoculated with a 10 μl culture of supernatant containing DENV (10 × LD_50_), which had been pre-incubated for 1 h at 37°C with serial dilutions of purified HMAb 1G5 (100, 10, or 1 μg/ml) or PBS (as a negative control). For the next 3 weeks, the mice were observed every day for signs of infection, such as ruffled hair, hunched back, paralysis, and death.

To evaluate the therapeutic efficacy of HMAb 1G5, a single dose of 5 μl containing 1 μg of the antibodies was intracerebrally administered at 6 h after challenge with 5 μl DENV-1 or DENV-2 (10 × LD_50_).

All animal experimental procedures were approved by the Animal Care and Use Committee of the Academy of Military Medical Sciences (AMMS) (ID:SYXK2012-05) and were carried out in strict accordance with the guidelines. All experiments involving the live virus were performed in an approved biosafety level 3 facility.

### ADE Assays

According to the findings of a previous report (Hezareh et al., 2001; Goncalvez et al., 2007), we introduced leucine-to-alanine mutations at positions 234 and 235 of IgG1 or deleted 9 amino acids (at positions 231–239) in the N terminus of the Fc domain to generate two plasmids encoding a mutant heavy chain. The plasmid encoding the light chain was representative of the wild-type antibody. As described previously, antibodies were expressed in FreeStyle^TM^293-F cells and purified by HiTrap MabSelect Xtra. The K562 cell line is a conventionally used model for investigation of ADE. We therefore used this model in our study to examine ADE caused by dengue infection. Seed stocks of the four DENV serotypes were diluted so that the multiplicity of infection (MOI) was 0.1, and the wild-type or mutant antibodies were added and incubated at 37°C for 1 h. The antibody solutions were diluted by four-fold by 12 serial dilutions from 30 μg/ml. K562 cells (2 × 10^5^) were incubated with the virus-antibody suspensions for 2 h at a temperature of 37°C. Following this, the K562 cells were washed two times with 1× PBS and resuspended in 1 ml of RPMI-1640 medium containing 2% FBS. The cells were incubated in this medium at 37°C in a 5% CO_2_ atmosphere for 4 days. Then, the cells were separated by centrifugation at 4000 *g*. The supernatant obtained was used to treat BHK-21 cells, with 100 μl of the supernatant added to each well (as the protocol of the PRNT assay); the experiment was performed in duplicate. The same viral strains that were used in the PRNT assay were used here too.

### Statistical Analysis

In the animal experiments, data were analyzed using GraphPad Prism software. The significance of the difference in the protective effect compared to the control group was evaluated using the Log-rank test. Differences with *P* values <0.05 were considered to be statistically significant.

## Results

### Generation and Identification of HMAbs Against DENV-1

We used FACS to isolate 1056 single plasma cells from 2 × 10^6^ PBMCs of patients who were confirmed to have naturally acquired DENV-1 infection in 2014 in Guangdong province. Then, 285 gene pairs of the obtained antibodies were amplified by PCR. The genes encoding heavy and light chains were cloned into different plasmid vectors that contained a human immunoglobulin constant and were expressed in FreeStyle^TM^293-F cells. Among the antibodies, 88 antibodies specific to DENV were isolated by an enzyme-linked immunosorbent assay (ELISA); the inactivated dengue virion was used as the coating antigen. To measure the neutralization potency of the 88 antibodies, the antibody concentration was fixed to 10 μg/ml and the corresponding plaque number was calculated. Twenty-four of the antibodies achieved a neutralization efficiency of 50% (**Supplementary Figure S2**).

### Characterization of HMAb 1G5

**FIGURE 1:**
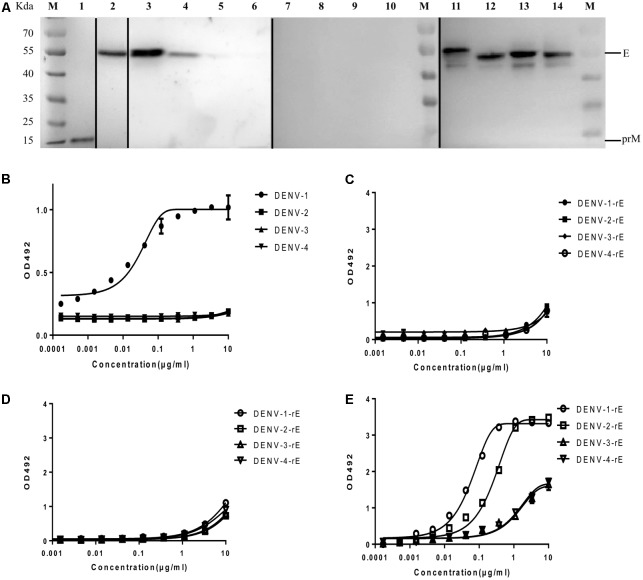
Binding of HMAb 1G5 to a conformational epitope on the E protein. **(A)** Virion non-reducing western blotting results for all four DENV serotypes. Lane M, molecular weight of the protein markers; lanes 1–3, 7, 11, DENV-1; lanes 4, 8, 12, DENV-2; lanes 5, 9, 13, DENV-3; lanes 6, 10, 14, DENV-4. All samples were obtained from the DENV-containing supernatant of C6/36 cells subjected to reducing (lanes 7–10) and non-reducing conditions (lanes 1–6 and 11–14). Antibodies used for detection: lane 1, DV69.6, which is a cross-reactive antibody against DENV that binds to the prM protein; lane 2, 4G2, which is a cross-reactive antibody that recognizes a conserved epitope present on the fusion loop of the flavivirus E protein; lanes 3–6,7–10, HMAb 1G5; lanes 11–14, 4G2. **(B)** ELISA results for the binding between HMAb 1G5 and the intact virion of the four DENV serotypes. 4G2 was utilized as a coating antibody for immobilization of non-inactivated virion. **(C–E)** ELISA results for the binding between HMAb 1G5 **(C)**, HMAb S4B11 **(D)**, HMAb 6B1 **(E)**, and the recombinant E protein of the four DENV serotypes. The assay plates were coated with the recombinant E proteins expressed by *E. coli*.

**FIGURE 2:**
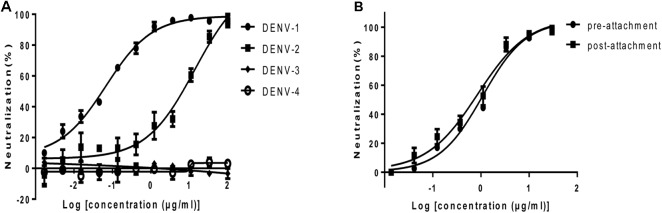
Neutralization activity of HMAb 1G5 in BHK-21 cells. **(A)** Neutralization of the four DENV serotypes by HMAb 1G5. DENV-1 and serially diluted HMAb 1G5 were incubated at 37°C for 1 h. Plaque reduction assays were used to evaluate the neutralization activity of HMAb 1G5 in BHK-21 cells. Results are shown as the percentage of plaque reduction. Data were obtained from three separate experiments and shown as the mean ± SD. **(B)** Neutralization efficiency of HMAb 1G5 against DENV-1 before and after attachment. The cells were incubated with serially diluted HMAb 1G5 1 h before or after DENV-1 infection. Plaque reduction assays were used to evaluate the neutralization activity of HMAb 1G5 in BHK-21 cells. The results are shown as the percentage of plaque reduction. Data were obtained from two separate experiments and shown as the mean ± SD.

It was shown that 10 μg/ml of HMAb 1G5 was able to neutralize approximately 100% of the DENV-1 viruses (**Supplementary Figure S2**), so we chose this HMAb for further research. In order to determine whether HMAb 1G5 recognized the prM protein or the E protein, western blot analysis was used to examine the reactivity of HMAb 1G5 to unheated and non-reducing virus samples. We found that HMAb 1G5 specifically recognized the E protein of DENV-1 and DENV-2, but it did not react with DENV-3 or DENV-4 (**Figure 1A**). More importantly, HMAb 1G5 reacted with the E protein of the non-reducing virus sample but not the reducing sample (**Figure 1A**). We also found that HMAb 1G5 recognized a specific epitope that is displayed on the DENV-1 virion (**Figure 2B**), but it did not recognize the recombinant E protein expressed by *E. coli* (**Figure 1C**). The non-related antibody did not recognize the recombinant E protein (**Figure 1D**), while the positive control antibody recognized it (**Figure 1E**).

### *In Vitro* Neutralizing Activity of HMAb 1G5

To examine the neutralization potential of HMAb 1G5, a standard plaque reduction neutralization assay (PRNT) was performed using BHK-21 cells. The antibody exhibited strong neutralization activity against DENV-1 and DENV-2. The PRNT_50_ values for DENV-1 and DENV-2 were 0.07 μg/ml and 13.84 μg/ml respectively (**Figure 2A**).

Attachment and fusion of the virus are important phases of viral infection. In order to investigate the step at which HMAb 1G5 inhibited DENV-1 infection in the host cells, we compared the neutralization profile of HMAb 1G5 against DENV-1 before and after viral attachment to cells. The neutralization efficiency was similar both before (PRNT_50_, 1.08 μg/ml) and after viral attachment (PRNT_50_, 0.85 μg/ml) (**Figure 2B**). These findings indicate that HMAb 1G5 neutralized DENV-1 mainly after the attachment step in the viral life cycle. Thus, HMAb 1G5 may be useful both in prophylactic treatment as well as therapeutic treatment of DENV-1 infection.

### *In Vivo* Protection Efficacy of HMAb 1G5

**FIGURE 3:**
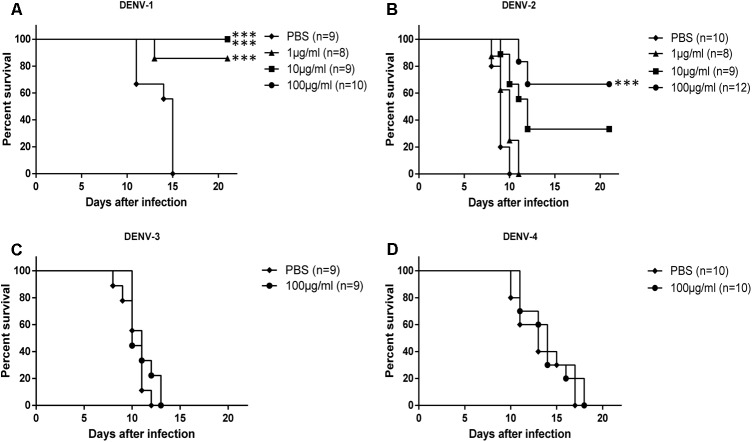
Protective effect of HMAb 1G5 against challenge with the four DENV serotypes in suckling mice. HMAb 1G5 was incubated with 10 × LD_50_ of DENV-1 **(A)** or DENV-2 **(B)** at a concentration of 100, 10, or 1 μg/ml and DENV-3 **(C)**, or DENV-4 **(D)** at a concentration of 100 μg/ml, then intracerebrally administered in the suckling mice. PBS was administered in the negative control. Mortality was determined in each group, and Kaplan–Meier survival analysis with the Log-rank test was performed (^∗∗∗^*p* < 0.001).

To analyze the *in vivo* protection efficacy of HMAb 1G5, an already reported suckling mouse model of DENV infection was used. In the control mice, the characteristic neurological symptoms appeared as quickly as at 5 days, and they died at 7–15 days after infection. The protection efficacy significantly differed between the four different serotypes of DENV (**Figure 3**). The protection efficacy of HMAb against DENV-1 and DENV-2 was observed to be dose dependent, and it was significantly better against DENV-1 than against DENV-2 at the same concentration. In particular, treatment with 100 μg/ml or 10 μg/ml HMAb 1G5 conferred complete protection against the DENV-1 challenge [10 × median lethal dose (LD_50_)], while treatment with 1 μg/ml HMAb 1G5 conferred protection in 85% of the mice. With regard to the DENV-2 challenge, treatment with 100 μg/ml HMAb 1G5 conferred protection in 67% of the animals, and survival was significantly higher than that in the control group (*P* < 0.001). All these findings show that while HMAb 1G5 protects against DENV-1 infection and provides partial protection against DENV-2, it does not confer protection against DENV-3 or DENV-4 infection.

**FIGURE 4:**
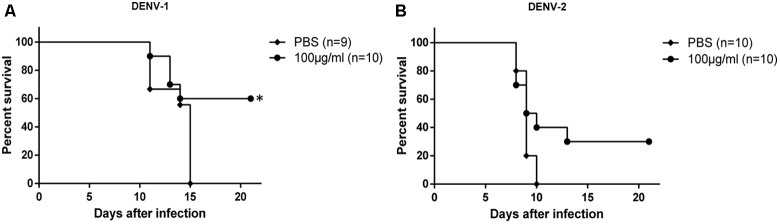
*In vivo* therapeutic effects of HMAb 1G5. A 1-μg dose of HMAb 1G5 was intracerebrally administered at 6 h after challenge with 10 × LD50 of DENV-1 **(A)** or DENV-2 **(B)**. PBS was administered as the negative control. Mortality was determined in each group, and Kaplan–Meier survival analysis with the Log-rank test was performed (^∗^*P* < 0.05).

Therapeutic efficacy of HMAb 1G5 was assessed by intracerebral administration of a single 1 μg dose at 6 h after challenge with DENV-1 or DENV-2 (10 × LD_50_). HMAb 1G5 conferred protection in 50% of the mice challenged with DENV-1 and 30% of the mice challenged with DENV-2 (**Figure 4**). These results demonstrate the *in vivo* therapeutic potential of HMAb 1G5 against DENV-1 infection.

### *In Vitro* Elimination of HMAb 1G5-Induced ADE

**FIGURE 5:**
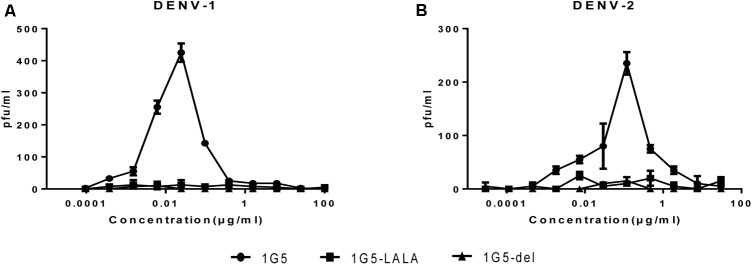
Elimination of HMAb 1G5-induced ADE with HMAb 1G5-LALA and HMAb 1G5-del. Serial dilutions of HMAb 1G5, HMAb 1G5-LALA, and HMAb 1G5-del were incubated with DENV1 **(A)** and DENV-2 **(B)** before they were added to K562 cells. After 4 days, plaque assay was used to quantify the amount of virus present in the supernatant of infected K562 cells. Data were obtained from two separate experiments and shown as the mean ± SD.

Studies have shown that administration of neutralizing antibody at sub-neutralizing concentrations enhances DENV infection in Fcγ receptor-positive cells. We investigated if this was true in the case of HMAb 1G5 by using the K562 cell enhancement assay, in which FcγRII-mediated uptake of virus-antibody complexes is used to induce infection. As expected, HMAb 1G5 induced ADE at a concentration of 0.1–0.001 μg/ml. In order to ensure the safety of using HMAb as a therapeutic agent, we tried to find a way of abrogating ADE caused by HMAb. According to previous studies, ADE can be eliminated by introducing Leu-to-Ala mutations (at positions 234 and 235) or deleting 9 amino acids (at positions 231–239) in the N terminus of the Fc domain (Hezareh et al., 2001; Goncalvez et al., 2007). We also used this method to create 1G5-LALA and 1G5-9 del and successfully abrogate ADE (**Figure 5**).

## Discussion

Cytofusion-hybridomas and EBV immortalized B cell lines (Lanzavecchia et al., 2007; Low et al., 2017) were earlier the most common sources of monoclonal antibodies, but they were associated with a low transformation efficiency (1–3%) (Aman et al., 1984; Borrebaeck et al., 1988; Crain et al., 1989). Later, Tiller developed a method to isolate single B cell-expressing clones from plasmablasts by FACS (Tiller et al., 2008). Using this method, Dejnirattisai et al. (2015) characterized 145 HMAbs from viremic patients infected with the DENV. Therefore, in the present study, we used FACS to isolate HMAbs from the plasma cells of infected patients. In the present study, we isolated single human plasma cells and amplified 285 genes pairs of antibodies by single-cell PCR of 1056 single cells. The efficiency of cloning Ig genes from single human plasma cells is 27%; this means that our method showed comparable efficiency (Tiller et al., 2008). However, only 88 out of the 285 antibodies were specific for DENV. Additionally, irrespective of their original isotype, all the antibodies here were generated as IgG1 molecules. Ideally, a part of the constant region should be amplified along with the variable region in order to discriminate between different isotypes.

In our present study, HMAb 1G5 displayed potent *in vitro* neutralizing activity and *in vivo* protective efficacy against DENV-1. The neutralizing activity of HMAb 1G5 was similar to that of HMAb 14c10 and 1F4 against DENV-1 (de Alwis et al., 2012; Teoh et al., 2012). However, HMAb 14c10 and 1F4 are specific for DENV-1, while HMAb 1G5 showed activity against DENV-2, although the neutralizing activity was not as strong as that against DENV-1. Thus, HMAb 1G5 seems to have a more broad spectrum of activity and also displayed potent protective efficacy against both DENV-1 and DENV-2. Altogether, the findings show that HMAb 1G5 may be a promising therapeutic agent against DENV-1.

We found that ADE was completely abrogated after modification of the Fc region of the antibody via amino acid deletion and leucine-to-alanine mutations. It has been reported that the prophylactic and therapeutic efficacy of antibodies with these modifications is not compromised (Goncalvez et al., 2007; Beltramello et al., 2010), and there was no significant reduction in their half-life too (Hessell et al., 2007; Leabman et al., 2013). These findings imply that the Fc modification is a feasible method for generating therapeutic antibodies against the DENV that do not induce ADE. However, these modifications could affect the other immune functions of the antibodies, such as those in the ADCC and complement pathways. Future studies will be required to investigate in more detail the potential of these modified antibodies as therapeutic agents in humans.

Most of the potent neutralizing HMAbs against specific DENV serotypes reported so far recognize structural epitopes that are only found on the intact virion (de Alwis et al., 2012). For example, DENV-1 specific HMAb 14c10 recognizes a quaternary epitope consisting of adjacent E protein dimers, and binding sites are present in all the three E domains (Teoh et al., 2012). Further, DENV-1 specific HMAb 1F4 recognizes a specific DI-II hinge angle; this epitope is present only when the E protein is displayed on the virion but not on the recombinant E protein (Fibriansah et al., 2014). Since our findings show that HMAbs 1G5 binds the E protein of intact DENV-1 or E protein under unheated and non-reducing conditions but not the recombinant E protein, we propose that HMAb 1G5 recognizes a specific conformational epitope. This epitope seems to be specific for DENV-1 and partially specific to DENV-2. However, further experiments are required to understand its specificity for different serotypes.

In conclusion, we have generated and characterized a novel DENV-specific HMAb 1G5 that binds to the E protein of only intact DENV-1 or the E protein under unheated and non-reducing conditions but not to recombinant E protein; the finding indicates that HMAb 1G5 recognizes a specific conformational epitope of the envelope protein of DENV-1. In addition, HMAb 1G5 was found to have a potent *in vitro* neutralization effect against DENV-1 infection and a strong *in vivo* protective effect in suckling mice. However, this neutralization effect was poor in the case of DENV-2 infection and was not observed against DENV-3 and DENV-4 infection. Finally, FcγRII-mediated uptake of immune complexes and by the introduction of Leu-to-Ala mutations (1G5-LALA) or deletion of nine amino acids (1G5-9del) in the Fc region of the antibody. Thus, HMAb 1G5 is safe and has potential for use in the prophylactic and therapeutic treatment of DENV-1 infection.

## Author Contributions

JL performed the experiments, analyzed the data, and wrote the manuscript. RW, BX, and YY analyzed the data and wrote the manuscript. PH, ZY, and XZ designed the study. All authors read and approved the final version of the manuscript.

## Conflict of Interest Statement

The authors declare that the research was conducted in the absence of any commercial or financial relationships that could be construed as a potential conflict of interest. The handling Editor declared a shared affiliation, though no other collaboration, with the authors.

## Footnotes

1 http://www.who.int/news-room/fact-sheets/detail/dengue-and-severe-dengue

2 http://www.nhfpc.gov.cn/jkj/s3578/201502/847c041a3bac4c3e844f17309be0cabd.shtml

## Supplementary Material

FIGURE S1 The constructs of expression plasmid vectors of HMabs. **(A)** pTSEG1n, **(B)** pTSEK, **(C)** pTSEL.

FIGURE S2 Identification of HMAbs against DENV-1. The concentration of the antibodies was fixed to 10 μg/ml, and the number of plaques formed by DENV-1 after treatment with each antibody was calculated to measure neutralization potency.
