# Supplementary figures and images for: Potent Neutralization Ability of a Human Monoclonal Antibody Against Serotype 1 Dengue Virus
Source: Front Microbiol. 2018 Jun 6;9:1214. doi: 10.3389/fmicb.2018.01214 (PMC5997965; doi:10.3389/fmicb.2018.01214)

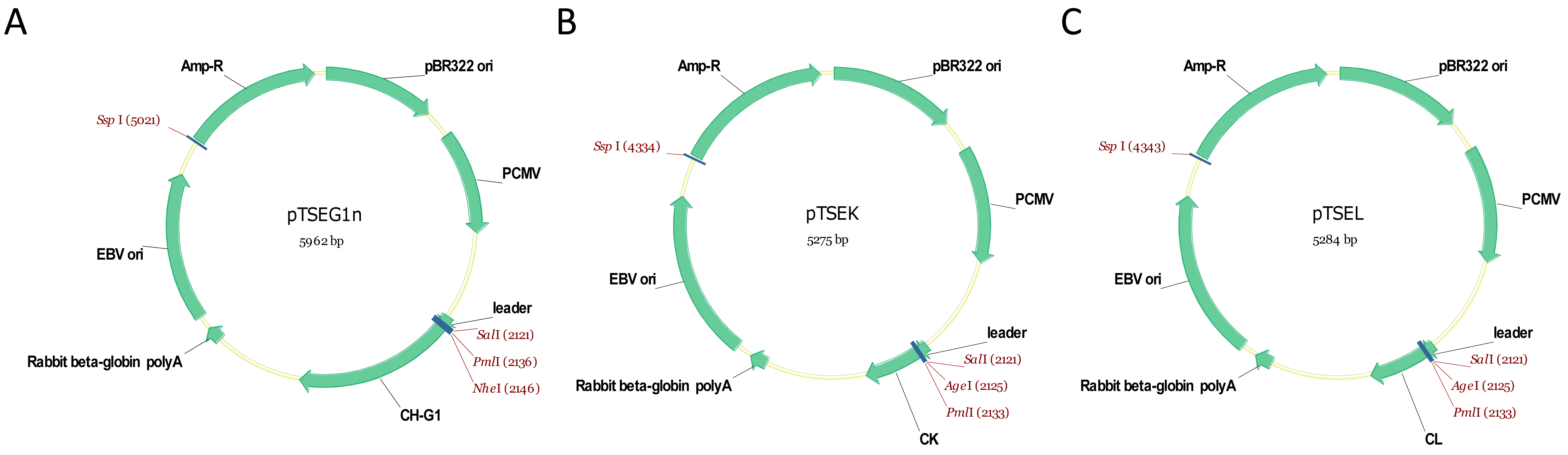

Supplement: FIGURE S1 — The constructs of expression plasmid vectors of HMabs. (A) pTSEG1n, (B) pTSEK, (C) pTSEL. [file Image_1.TIF]

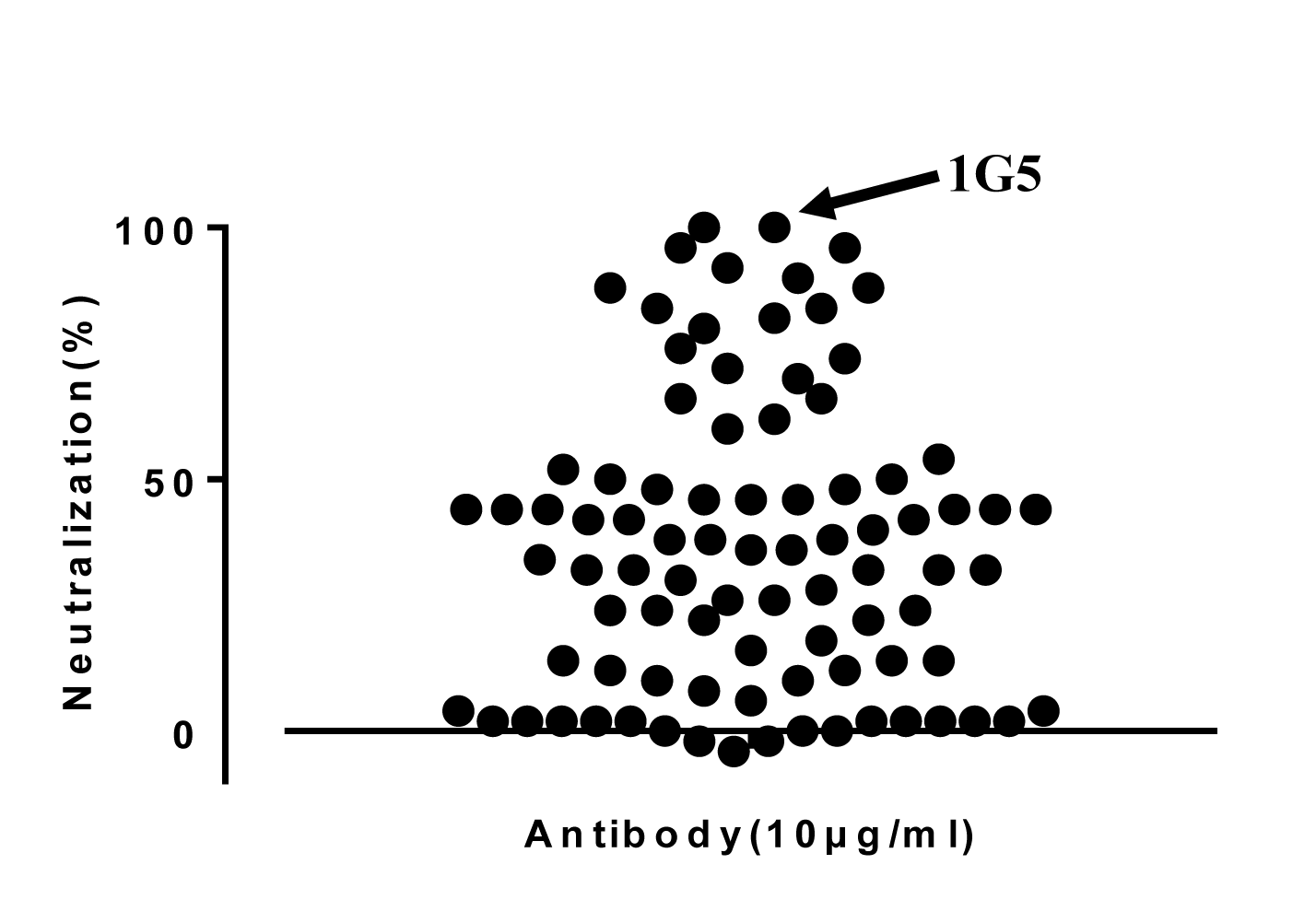

Supplement: FIGURE S2 — Identification of HMAbs against DENV-1. The concentration of the antibodies was fixed to 10 μg/ml, and the number of plaques formed by DENV-1 after treatment with each antibody was calculated to measure neutralization potency. [file Image_2.TIF]
